# Influence of Probiotics in Prevention and Treatment of Patients Who Undergo Chemotherapy or/and Radiotherapy and Suffer from Mucositis, Diarrhoea, Constipation, Nausea and Vomiting

**DOI:** 10.3390/jcm11123412

**Published:** 2022-06-14

**Authors:** Aleksandra Garczyk, Iwona Kaliciak, Konstanty Drogowski, Paulina Horwat, Stanisław Kopeć, Zuzanna Staręga, Paweł Bogdański, Marta Stelmach-Mardas, Marcin Mardas

**Affiliations:** 1 Department of Treatment of Obesity, Metabolic Disorders and Clinical Dietetics, Poznan University of Medical Sciences, Szamarzewskiego Street 84, 60-569 Poznan, Poland; garczykaleksandra@gmail.com (A.G.); ikaliciak@o2.pl (I.K.); drogowskos@wp.pl (K.D.); paulina.horwat9@gmail.com (P.H.); s.kopec@macron.pl (S.K.); zuziastarega@icloud.com (Z.S.); pbogdanski@ump.edu.pl (P.B.); stelmach@ump.edu.pl (M.S.-M.); 2 Department of Gynecological Oncology, Institute of Oncology, Poznan University of Medical Sciences, 60-569 Poznan, Poland

**Keywords:** chemotherapy, radiotherapy, probiotics, mucositis, diarrhoea, constipation, nausea, vomiting

## Abstract

The administration of probiotics to patients treated with chemo- and/or radiotherapy is assumed to be beneficial. The aim of this study was to evaluate the effects of probiotic intake on the severity of selected gastrointestinal side effects of chemotherapy and radiotherapy. The searched databases included PubMed, Web of Science, and Scopus from which twenty-one studies were included. Most of them concerned diarrhoea, however, two of the studies examined constipation, another two nausea and vomiting, and eight of the included studies regarded mucositis. The total number of patients equalled 2621. The time of the conducted therapy, the administered species, neoplasm pathology, and adjuvant therapy varied. The outcome was assessed by gathering information about the statistical significance of the improvements. An enhancement was observed in thirteen studies, where probiotics had a significant impact on each of the included chemo- and/or radiotherapy side effects. However, the heterogeneity of the assessed data makes it impossible to state a firm conclusion.

## 1. Introduction

Huge progress made in the treatment of cancer is not accompanied by the development of methods to prevent the unpleasant side effects of therapy [1,2]. According to the American Society of Clinical Oncology (ASCO) and the European Society for Medical Oncology (ESMO) guidelines [3,4,5], the most commonly chosen therapeutic options are radiotherapy and chemotherapy, as a monotherapy or as an adjuvant therapy with surgical intervention. The cells of the gastrointestinal epithelium are mainly affected by the cytotoxic effect of these therapeutic agents [6]. Therefore, the side effects related to chemotherapy or radiotherapy directly influence the digestive system. Usually, patients suffer from mucositis, diarrhoea, constipation, nausea, and vomiting [7]. Alleviation of them is crucial to the improvement of patients’ comfort as they strongly influence therapy results [2,8]. It was suggested that probiotic administration may help to achieve this goal [9], particularly as they have shown a protective effect on epithelial cells [10]. The bacteria in the genus *Lactobacillus* are the most frequently studied and are considered to be possibly related to the reduction of undesirable effects [9,10,11,12]. Additionally, research on *Bifidobacterium* strains indicates promising outcomes for oncological patients [11,13]. However, there is limited available data on the effectiveness of probiotics, especially when it comes to constipation, mucositis, nausea, and vomiting.

The aim of this study was to evaluate the effects of probiotic intake on the severity of chemotherapy and radiotherapy selected gastrointestinal side effects.

## 2. Materials and Methods

### 2.1. Search Strategy, Inclusion, and Exclusion Criteria

From September 2020 to December 2021, the following databases were searched and reviewed in order to identify interventional studies that investigate the influence of probiotics on chemotherapy- and/or radiotherapy-induced mucositis, diarrhoea, constipation, nausea, and vomiting: PUBMED (MEDLINE), SCOPUS, and WEB OF SCIENCE. The review was registered on the PROSPERO database (ID: CRD42021248256).

The search was limited to studies concerning humans and published in English. Original articles were included. No restrictions regarding the date of the publication or kind of neoplasm were used. Administering probiotics orally was a required inclusion criterion. Taking into account the study design, the following articles were included: randomised, double-blind, placebo-controlled study (RDBPC) (10), randomised controlled study (RCT) (9), and non-randomised controlled study (NRS) (2). The articles with low-quality or incomplete data that could not be fully obtained from the authors were excluded.

The search strategy included the following index terms: *#1 probiotics OR probiotic OR probiotic bacterium OR probiotic microflora OR probiotic flora; #2 mucositides OR mucositis OR inflammation of mucosa OR mucous membranes inflammation OR mucous membrane inflammation OR mucosal inflammation OR mucosal lesions OR diarrheas OR diarrhoea OR diarrhea OR obstruction OR constipation OR dyschezia OR colonic inertia OR nausea OR emesis OR vomiting OR CINV OR breakthrough CINV OR anticipatory CINV; #3 Neoplasms OR Neoplasms OR Neoplasia OR Neoplasias OR Neoplasm OR Tumors OR Tumor OR Malignant Neoplasms OR Malignant Neoplasm.*

*#1 AND #2 AND #3.*

### 2.2. Data Extraction and Analysis

Three different teams separately reviewed the databases in order to identify articles that fulfilled the inclusion criteria. Each team consisted of two independent researchers. Firstly, titles and abstracts were screened by every researcher independently. Secondly, a detailed assessment of the full texts was conducted by each team in order to select eligible articles. Lastly, the selected studies were jointly evaluated by all researchers, who decided on the inclusion or exclusion of a study.

Included studies were appraised in order to derive the title, main author, publication year, study name and design, countries involved, total number of patients, age, sex, and type of neoplastic disease and its staging. From the medical interventions, the following information was obtained: species included in the probiotic, method of probiotic administration, dosage, and duration of treatment. Moreover, the occurrence of adverse effects related to probiotics was assessed. The implemented treatment details about both chemo- and radiotherapy and any additional therapies were extracted.

The following definitions of selected gastrointestinal side effects were used in the data interpretation of the single studies. Diarrhoea is defined as loose or semisolid discharge, which appears at least three times per day or more often than usual [14]. The assessment of diarrhoea comprises grades according to the National Cancer Institute Common Toxicity Criteria (NCI CTC) 2.0, NCI CTC 3.0, the Common Terminology Criteria for Adverse Events (CTCAE) 4.0, CTCAE 4.1, or the World Health Organisation’s (WHO) duration, frequency, and consistency degrees according to the Bristol scale, and abdominal discomfort occurrence and administration of antidiarrheal medications. Constipation is described as less than three bowel movements per week associated with other symptoms such as hard stools, bloating, distention, abdominal discomfort, excessive straining, a feeling of anorectal blockage, and incomplete defecation [15]. Constipation characteristics include the duration, frequency, and the Wexner score, which describes faecal incontinence. Nausea is a subjective feeling, which might precede vomiting [16]. Vomiting is an expeditious removal of gastric contents through the mouth [16]. For nausea and vomiting, information on the duration and grade according to CTCAE 3.0 was selected. Mucositis is defined as the erythema and ulceration of the gastrointestinal tract [17]. Concerning mucositis, the following data were extracted: grade according to the NCI CTC, time to onset, time to resolution or healing, and the administration of additional nutrition.

## 3. Results

### 3.1. Search Results

**Figure 1:**
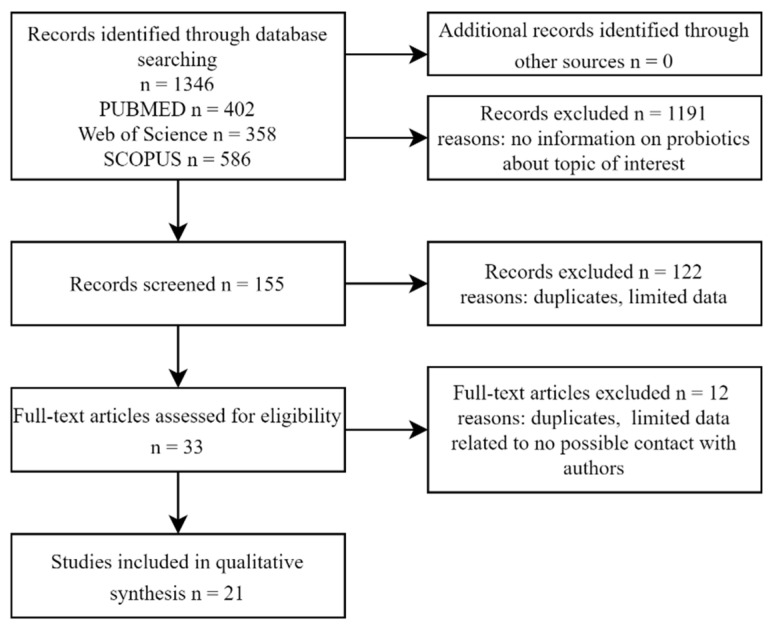
Flow chart of the database searches on influence of probiotics on radio- and chemotherapy side effects.

The flow chart of the database searches is shown in Figure 1. A total of 1346 articles were selected as a result of the screening of the studies’ titles. The assessment of the studies’ abstracts led to the exclusion of 1191 papers. Another 122 positions were removed after consultations due to insufficient data about probiotics used during intervention. Finally, the full texts of 33 articles were carefully examined, with 12 studies being eliminated for incomplete information about changes in the condition of patients during probiotic intake as well as for no possible contact with the authors. A total of 21 papers met the inclusion criteria and were involved in the qualitative synthesis.

### 3.2. Characteristics of the Included Studies and Study Population

Information about the characteristics of the included studies is presented in Table 1. From twenty one studies ten studies were randomised double-blind control trials (RDBCT) [18,19,20,21,22,23,24,25,26,27] which is considered the “Gold Standard” in intervention-based studies. The studies were conducted mainly on European [18,22,23,24,28,29,30,31] and Asian [19,20,21,25,27,32,33,34,35,36,37] populations. The total population consisted of 2619 individuals. Patients were administered probiotic mixtures with a defined composition. In one study [34], probiotic kefir was applied. Combinations of bacterial strains from the *Lactobacillus*, *Streptococcus,* and *Bifidobacterium* genera were administered simultaneously [18,19,21,23,24,25,26,27,29,31,32,33] 2–3 times per day. The time of the intervention varied and it was shorter than 8 weeks in most of the studies [18,19,20,21,22,25,27,30,32,33,36,38]. However, it lasted for 24 weeks in one of them [28].

The detailed characteristics of the study population are shown in Table 2. Most of the studies were conducted in adult populations [18,19,20,21,22,23,24,25,26,27,28,29,30,31,32,33,34,36]. Out of 21 studies, 3 covered the paediatric population [35,37,38]. Colorectal and cervix cancers [18,19,21,22,24,26,28,29,32,34,36] were the most common diagnosis for older patients, whereas among children the most widespread neoplasms were lymphomas [35,38], leukemia [35,38], and central nervous system tumours [37]. The vast majority of patients suffered from an advanced stage of tumour. The therapy that the patients underwent was dependent on the type of tumour. Chemotherapy conducted among patients with cervical cancer and head and neck tumours included cisplatin [18,20,21,25,26,27,30]. Lower abdominal neoplasms were treated mainly by 5-fluorouracil [24,28,34]. The mean duration of implemented radiotherapy was 5.6 weeks [18,20,21,22,23,24,25,26,28,29,30,31,32,33,34,35,36,37,38]. In 8 out of 21 studies [18,19,23,24,28,29,31,37], invasive anti-cancer treatments including surgery were performed. Patients were administered antiemetic drugs, antidiarrhoeal drugs, antianalgesic drugs, antibiotics, and antifungal drugs when needed.

### 3.3. Effects of Interventions

A summary of the outcomes is presented in Table 3, Table 4, Table 5 and Table 6. The conditions of the patients who qualified for the study and control groups were compared in four categories: diarrhoea, constipation, nausea and vomiting, and mucositis. The outcomes were described as improvements or non-significant results. Improvement means at least one significant outcome. The studies regarded different parameters in order to assess the significance of the differences between the study and control groups.

Ailments related to diarrhoea after receiving probiotics during chemotherapy and/or radiotherapy were remarkably less severe [19,21,23,28,31,32,33,37,38], which was presented in Table 3. The occurrence of diarrhoea was limited in 6 out of 15 studies [19,24,29,31,37,38]. The degree of diarrhoea was assessed by different scales. The NCI criteria were used [18,19,21,24,28,32] as well as the WHO scale [23,26,29]. One study assessed diarrhoea using the investigator’s scale [22] and information regarding the used criteria were missing in [31]. The percentage of patients with a higher degree of diarrhoea significantly decreased in the groups receiving probiotics compared to the controls [19,21,23,28,29,31,32,37]. The duration of diarrhoea was minimised after probiotic intake [35,38]. Also, the number of daily incidents of diarrhoea decreased [22,23,26,31,32,35]. The assessment of stool consistency (Bristol scale) in patients who suffered from diarrhoea was performed in four studies [18,21,26,32]. Three studies used a simplified scale in order to facilitate its use by patients [21,26,32]. However, a full 7-points scale was also used by patients [18] and in one study the investigator’s scale was used [22]. The results of the stool consistency examination are contradictory. In three out of five studies [21,22,32], a lower number of looser stools was noticed in the study groups. In other studies [18,26], differences were not observed. Moreover, shorter and less frequent incidences of abdominal pain [19,33] and abdominal discomfort (regarded as flatulence, borborygmia, or distension) [28] were observed in the study groups. However, more severe abdominal discomfort (described as bloating) was found in one study [32] after probiotic intake. Most of the studies recorded the use of antidiarrheal drugs [19,21,22,23,24,26,31,32], except for [32], which did not specify the drugs used, and loperamide was used in [18,19,21,22,23,24,26,28,31]. Most patients in the study groups were characterised as having less need for the admission of antidiarrheal drug [19,21,22,23,24,26,31,32]. However, the outcomes were significant in four out of eight studies [21,23,31,32].

In studies that analysed constipation [36,38] as well as nausea and vomiting [37,38], probiotic therapy has been recognised as a factor that could have a beneficial impact. Receiving probiotics was accompanied by lower scores in the Wexner classification [36] and a shorter duration of constipation [38] (Table 4). However, only the results regarding the duration of constipation were significant [38]. Additionally, significant improvements in stool character and frequency were observed in [36]. A reduction in the duration of vomiting [38] and the nausea grade according to CTCAE 3.0 [37] in the study groups compared to the control groups was regarded as significant.

In most of the studies that focused on mucositis, an alleviation of symptoms following probiotic intake was reported [20,25,27,37]. The results regarding the differences in mucositis between the study and control groups varied widely. No significant differences in probiotic treatments were observed in two out of six studies [30,34]. The severity of mucositis was significantly lower in two studies [20,25,27]. The toxicity of the treatment, regarded as mouth erythema or ulcers, was also significantly alleviated in [37]. In one study [30], enteral nutrition was applied in the study group, which is considered to be a determinant for an improvement in mucositis. Also, parenteral nutrition was administered and a Ryle’s tube was inserted when needed [20]. The requirement for parenteral nutrition or a Ryle’s tube was significantly lower for the study group [20].

## 4. Discussion

According to the collected data, the influence of probiotics on the severity of chemo- and/or radiotherapy side effects may not be established. Furthermore, relevant uncertainties regarding the administration of probiotics also occur.

There were no incidences of adverse effects confirmed as a result of the administration of probiotics. Nevertheless, it needs to be mentioned that patients undergoing chemo- and/or radiotherapy are at greater risk of adverse effects induced by probiotics than the healthy population. Therefore, it is highly recommended to observe patients who receive probiotics [39]. There are cases of bacteraemia caused by *Lactobacillus* strains (such as *L.* GG, *L. casei* strains, *L. acidophilus* strains), *Bacillus* species (*B. subtilis*), and *Bifidobacterium* species (*B*. *breve)* [39]. The development of abscesses after *L. rhamnosus* [39] and *L. GG* [40] administration as well as endocarditis caused by *L. GG* and *L. rhamnosus* [40] were also reported. The safety of probiotics administered to patients suffering from neoplasms is not established [39,40,41].

Concerning the administration of probiotics, the variety of the chosen species needs to be taken into account as a factor that influences the outcome. Similarly, the dosage of probiotics and the duration of the treatment differed. To our knowledge, there are no recommendations regarding both dosage and time of intervention while undergoing chemo- and/or radiotherapy. Nonetheless, in most of the included studies treatment started on the first day of chemo- and/or radiotherapy [19,20,23,25,26,27,28,29,30,31,36]. Only in four studies were probiotics administered at least 7 days prior to the beginning of therapy [18,21,32,35]. It is possible that therapies could diminish the protective effects of probiotics due to the limitations of their prophylactic possibilities, which could be overcome by administering probiotics one month prior to therapy [9]. Moreover, the influence of probiotic administration on the microbiome should be considered as an observation of the differences between the placebo and control groups [25]. Additionally, there is a possibility that probiotic administration could be helpful in balancing gut dysbiosis during cancer treatment [27].

It is assessed that changes to the human microbiome as a result of treatment [42] have a huge impact on the development of digestive system-related chemo- and/or radiotherapy side effects. Anti-cancer therapy can lead to a reduction in gut bacteria diversity and, more importantly, to a decrease in bacteria that limit inflammation and increase bacteria associated with mucositis [43]. Furthermore, the polyamine transport deficiencies associated with the increased risk of cytotoxic T cell antigen 4 (CTLA-4) blockade-induced colitis may be caused by this disruption to the ecological network balance in the gastrointestinal tract [44]. Probiotic intake greatly corrects the composition of the microbiome [45], which could be beneficial for a reduction in side effects. Another mechanism resulting in disorders in the gastrointestinal tract is the activation of transcription factors, particularly nuclear factor-κB (NF-κB) and the subsequent upregulation of pro-inflammatory cytokines and inflammatory mediators [46]. *B. bifidum*, *B. longum*, *B. longum* subsp. *infantis,* and *L. rhamnosus* may have the potential to reduce tumour necrosis factor α (TNF-α) and interleukin 1β (IL-1β) concentrations [47], whereas a decrease in NF-κB, interleukin 6 (IL-6), and TNF-α is observed during *L. delbruekii* and *L. fermentum* administration [48]. The main mechanism with which the therapy fights cancer cells is the induction of apoptosis. However, it also applies to other cells, especially those that undergo rapid proliferation such as gastrointestinal epithelial cells [49]. *B. lactis* reduces apoptosis and improves cellular renewal by encouraging proliferation among intestinal cells [50]. *L. rhamnosus GG* and a few other strains are also known to have similar properties [51]. An important role in the initial phase of the mucositis process is attributed to the generation of reactive oxygen species [52]. The administration of *Escherichia coli* successfully prevents lipid peroxidation and the decline of mucosal glutathione [53]. Also, multistrain probiotic VSL#3 (*L. plantarum*, *L. acidophilus*, *L. casei*, *L. delbrueckii* subsp. *bulgaricus*; *B. infantis*, *B. breve*, *B. longum*, *S. salivarius* subsp. *thermophilus*) is known to have a positive effect on the extent of glutathione and additionally can reduce the expression of inducible nitric oxide synthase, protein nitrosylation, and malondialdehyde levels in rats [54]. This leads to a highly antioxidative effect. The growth of intestinal permeability above the average is another component of mucosal impairment. It is followed by an increased risk of the expansion of pathogens and a general loss of intestinal functions [55]. Tight junctions (TJ), which consist of occludin, claudin, and the zonula occludens (ZO) family of proteins, among others, are sealing the space between the epithelial cells and regulating their permeability. *Escherichia coli* intake is leading to increased expression of ZO-2 protein and a redistribution of ZO-2 from the cytosol to the cell boundaries. Furthermore, *B. infantis* Y1 produces substances that lead to increased ZO-1 expression [56] and the administration of *B. infantis* Y1 and *L. plantarum* increases occluding protein expression [56]. This provides a protective effect against chemically induced destruction of the epithelial barrier [56].

The effectiveness of probiotics during chemo- and/or radiotherapy-induced diarrhoea was observed in most of the included studies [19,21,23,28,31,32,33,37,38] despite some variances, which appears consistent with the conclusions of other studies [9,41,57,58,59,60,61]. Nevertheless, patients were suffering from different neoplasms and, therefore, therapy might have been focused on only one region of the body. Regarding abdominal and pelvic neoplasms, which occurred in most of the studies [18,19,21,22,23,24,26,28,29,31,32,33,34], it is well-established that probiotics have a beneficial influence [9,41,58,59,60]. However, the impact of probiotics on diarrhoea observed during therapy for both central nervous system neoplasms [37] and white blood cell neoplasms [35,38] is not properly described. Therefore, significant improvements in these two areas [35,38] should be made. What is more, the type of implemented therapy might have had an impact on the effectiveness of probiotics as it is firmly associated with neoplasm pathology. Moreover, the administration of probiotics might even be unrecommended for the treatment of diarrhoea during both chemo- and radiotherapy and for the prevention of diarrhoea during chemotherapy due to inconclusive data [61]. In contrast, in one of the included studies, the implementation of probiotics during individual chemotherapy treatment was found to be beneficial [38]. Similarly, the administration of probiotics during radiotherapy treatment was found to be effective [32]. Due to the diversity of the results, it is essential to conduct further research.

A positive impact on the condition of patients with mucositis after chemo- and/or radiotherapy that was reported in enrolled studies [20,25,27,37] confirms previous findings in the literature [62]. It is worth emphasizing that the results might have been strongly influenced by the composition of the probiotics. In most of the studies, the administered probiotics included *Lactobcillus* [20,25,27]. However, different species were considered in every study: *L. brevis* [20], *L. lactis* [25], *L. plantarum, L. rhamnosus,* and *L. acidophilus* [27]. What is more, Sharma et al. [20] reported the beneficial influence of the intake of only *L. brevis*, which correlates fairly well with [11] and further supports the concept that the bacteria from this family play a major role in alleviating inflammation. However, other species, such as *B. adolescensis* [33], which are considered beneficial for inflammation [62], were used in selected papers. *Lactobacillus* and *Bifidobacterium* were the most commonly used types of bacteria in articles, and they presented improvements in other ailments (diarrhoea, nausea and vomiting, constipation) [19,21,23,31,33,36] as well. This indicates the advantageous results of such a combination. However, different species were used in these studies. Moreover, radiotherapy induces changes in the intestinal microbiome, which might be balanced during probiotic administration [25].

There was little research related to constipation, however *B. tetragenous viable* was previously proven to be beneficial for constipation [36]. Our study provides further evidence for the effectiveness of probiotics during chemo- and/or radiotherapy. A decrease in the duration of constipation [38] and less severe ailments [36] were noted through the use of compositions containing *L. acidophilus*, *B. infantis* [36], and *L. rhamnosus* [38].

For the treatment of nausea and vomiting, traditional antiemetics are mainly used [63]. Our results offer evidence for the legitimacy of using probiotics for this purpose [37,38].

The most remarkable result to emerge from the data is that probiotics might be effective in treating a wide variety of ailments caused by radio- and/or chemotherapy. According to available knowledge, probiotics inhibit inflammation, maintain intestinal permeability, eliminate pathogenic bacteria, inhibit cell apoptosis, prevent oxidative damage, and maintain mucous barriers [64], which allows them to act comprehensively in preventing and curing the side effects of radio- and/or chemotherapy that are related to the digestive system.

## 5. Limitations

This study has distinct limitations. Firstly, not all the results were considered significant [18,22,24,26,30,34,35], which might indicate the positive effects of probiotics. Therefore, the outcomes of these studies were not able to evince the conclusion and have limited the number of studies taken into account. Secondly, insufficient data concerning constipation [36,38] and nausea and vomiting [37,38] were found. The limited description of these adverse effects prevented a thorough analysis. Moreover, it was impossible to generalise the research findings due to the heterogeneity of data. This prevented a firm conclusion and decreased the relevance of the outcomes of this review, which therefore must be interpreted with caution and the number of limitations should be considered. Regarding the involved studies, several limitations also occurred. Probiotics were administered in varying schedules, amounts, and dosages. The time of day when the probiotics were administered and the presence of meals before or after administration could have had an additional impact on the outcomes. Unfortunately, there are no regulations regarding this application. Additionally, probiotics are often prepared without following pharmaceutical standards, which could have caused insufficient responses [65]. Certainly, the additional intake of medication could also have had an impact on the outcomes, especially antiemetics, antidiarrheals, and analgesics, which could have influenced the responses to the interventions. Nevertheless, it is impossible to guarantee a drug-free trial that also considers the adverse effects of chemo- and/or radiotherapy.

In order to avoid the listed obstacles, it is necessary to provide well-designed trials and to ensure a detailed description of all regarded side effects. Moreover, the accurate assessment of live organisms included in probiotics should be assured.

## 6. Conclusions

The administration of probiotics has a positive influence on the condition of patients receiving chemotherapy and/or radiotherapy. The intake of probiotics leads to the alleviation of side effects such as diarrhoea, constipation, nausea, vomiting, and mucositis. Further research into the exact dosage, composition, timing of administration and safety of probiotics are needed.

## Figures and Tables

**Table 1:** Characteristics of the included studies (*n* = 21).

Study	Year	Country	Study Design	Study Population		Probiotics (Species, Components)		Probiotics (Daily Administration)		Time of Intervention (Weeks)
				Study Group	Control Group	Study Group	Control Group	Study Group	Control Group	
**ADULT POPULATION**										
**Chemotherapy treatment**										
Mego M. et al. [24]	2015	Slovakia	RDBPC ^1^	23	23	*Bifidobacterium breve* HA-129 (25%), *Bifidobacterium bifidum* HA-132 HA (20%), *Bifidobacterium longum* HA-135 (14.5%), *Lactobacillus rhamnosus* HA-111 (8%), *Lactobacillus acidophilus* HA-122 (8%), *Lactobacillus casei* HA-108 (8%), *Lactobacillus plantarum* HA-119 (8%), *Streptococcus thermophilus* HA-110 (6%), *Lactobacillus brevis* HA-112 (2%), *Bifidobacterium infantis* HA-116 (0.5%) 10 × 10^9^ CFU ^2^ per capsule, inulin, maltodextrin, magnesium stearate, ascorbic acid	inulin, maltodextrin, magnesium stearate, ascorbic acid	capsule p.o.^3^ 3 times a day	capsule p.o.	12
Liu J. et al. [36]	2014	China	RCT ^4^	50	50	*Bifidobacterium infantis*, *Lactobacillus acidophilus*, *Enterococcus faecalis*, *Bacillus cereus*	no intervention	capsules (4) p.o. 3 times a day	no intervention	4
**Radiotherapy treatment**										
Urbancsek H. et al. [22]	2001	Hungary	RDBPC	102	103	*Lactobacillus rhamnosus* 1.5 × 10⁹ CFU (1.5 g)	700 mg corn starch, 797 mg microcrystalline cellulose, 1.37 mg iron oxide, 1.13 mg dispersed orange, 1 mg caramel aroma	sachet p.o. 3 times a day	sachet p. o. 3 times a day	up to 1 (depending on the response of the diarrhoea)
Mansouri-Tehrani H.S. et al. [32]	2016	Iran	RCT	22	24	*Lactobacillus casei* 1.5 × 10^9^ CFU, *Lactobacillus acidophilus* 1.5 × 10^10^ CFU, *Lactobacillus rhamnosus* 3.5 × 10^9^ CFU, *Lactobacillus bulgaricus* 2.5 × 10^8^ CFU, *Bifidobacterium breve* 1 × 10^10^ CFU, *Bifidobacterium longum* 5 × 10^8^ CFU, *Streptococcus thermophilus* 1.5 × 10^8^ CFU (500 mg)	corn starch 500 mg	capsule p.o. 2 times a day (second one with yogurt)	capsule p.o. 2 times a day	5
Delia P. et al. [23]	2007	Italy	RDBPC	243	239	*Lactobacillus casei*, *L. plantarum*, *L. acidophilus*, *L. delbrueckii* subsp. *bulgaricus*, *Bifidobacterium longum*, *B. breve*, *B. infantis*. *Streptococcus salivarius* susp. *Thermophilus* 450 billions/g of viable lyophilized cells	N/A composition of placebo	sachet p.o. 3 times a day	sachet p.o.	from the start of RT ^5^
Delia P. et al. [29]	2002	Italy	RCT	95	95	*Lactobacillus casei*, *L. plantarum*, *L. acidophilus*, *L. delbrueckii* subsp. bulgaricus, *Bifidobacterium longum*, *B. breve*, *B. infantis*, *Streptococcus salivarius* susp. *thermophilus*	no intervention	bag p.o. 3 times a day	no intervention	N/A
Delia P. et al. [31]	2002	Italy	RCT	95	95	*Lactobacillus casei, L. plantarum, L. acidophilus, L. delbrueckii subsp. bulgaricus*, *Bifidobacterium. longum, B. breve, B. infantis*, *Streptococcus salivarius* susp. *Thermophilus* 450 billions/g of viable lyophilized	N/A composition of placebo	p.o. 3 times a day	p.o.	from the start of RT to finish cycle of RT
Shao F. et al. [33]	2013	China	RCT	24	22	*Bifidobacterium adolescent* is 0.5 × 10^9^, *Lactobacillus*, *Streptococcus thermophilus*	500 mL Peptiosorb solution (1 cal): 16% protein, 9% fat, 75% carbohydrates/mL)	capsules p.o. 3 times a day	p.o. 1 time a day	2
**Radiotherapy and chemotherapy treatment**										
Giralt J. et al. [18]	2008	Spain	RDBPC	44	41	*Lactobacillus* *casei* DN-114 001 108 CFU/g, in addition to the standard starters *Streptococcus* *thermophilus*, *Lactobacillus delbrueckii*, subsp. *bulgaricus*	sterilised active product with 4 kGy for 5 min	96 mL of fermented liquid yoghurt p.o. 3 times a day	96 mL p.o. 3 times a day	5–6
Ye-Htut-Linn et al. [19]	2017	Myanmar	RDBPC	26	28	*Lactobacillus acidophilus* LA-5, *Bifidobacterium animalis* subsp. *lactis* BB-12 1.75 × 10^9^ lyophilized live	starch	capsule with yogurt p.o. 3 times a day	capsule p.o. 3 times a day	5
Österlund P. et al. [28]	2007	Finland	RCT	98	52	*Lactobacillus rhamnosus* GG 1–2 × 10^10^	no intervention	gelatine capsule p.o. 2 times a day	no intervention	24
Sharma A. et al. [20]	2011	India	RDBPC	93	95	*Lactobacillus brevis* CD2 minimum 2 × 10^9^ viable cells	mixture of sugars and salts	lozenge p.o. 6 times a day	lozenge p. o.	8
Chitapanarux et al. [21]	2010	Thailand	RDBPC	32	31	*Lactobacillus acidophilus* minimum 10^9^, *Bifidobacterium bifidum* minimum 10^9^ (250 mg)	magnesium stearate, talc, purified water	capsules (2) p.o. 2 times a day	capsules p. o. 2 times a day	7.3
Topuz E. et al. [34]	2008	Turkey	NRS ^6^	17	20	250 mL of kefir	0.09% NaCl	oral lavage 2 times a day	oral lavage 2 times a day	N/A
de Sanctis V. et al. [30]	2019	Italy	RCT	32	36	*Lactobacillus brevis* CD2 2 × 10^9^ viable cells	sodium bicarbonate	lozenge p.o. 6 times a day	mouthwash 3 times a day	from the start of the RT to 1 week after
Jiang C et al. [25]	2018	China	RDBPC	58	35	*Bifidobacterium longum, Lactobacillus lactis*, *Enterococcus faecium*	starch	capsules (3) p.o. 2 times a day	capsules (3) p.o. 2 times a day	up to 7
Demers M et al. [26]	2014	Canada	RDBPC	standard dose 91 high dose 64	91	*Lactobacillus acidophilus* LAC-361, *Bifidobacterium longum* BB-536 standard dose 1.3 billion CFU high dose 10 billion CFU	N/A	capsule p.o. standard dose 2 times a da high dose 3 times a day	N/A	from the start of RT to the end of RT
Xia C. et al. [27]	2021	China	RDBPC	36	34	*Lactobacillus plantarum* MH-301 10^9^ CFU, *Bifidobacterium animalis* subsp. *Lactis* LPL-RH 10^9^ CFU, *Lactobacillus rhamnosus* LGG-18 10^9^ CFU, *Lactobacillus acidophilus* 10^9^ CFU	N/A	capsule p.o. 2 times a day	p.o. 2 times a day	6–7
**CHILD POPULATION**										
**Chemotherapy treatment**										
Reyna-Figueroa J. et al. [38]	2019	Mexico	RCT	30	30	*Lactobacillus rhamnosus* GG 5 × 109 CFU, maltodextrin	N/A	sachet p.o. 2 times a day	N/A	up to 1 (upon completion of: a 7-day probiotic course/chemotherapy/neutropenia onset)
Wada M. et al. [35]	2009	Japan	RCT	18	22	10^9^ freeze-dried, live *Bifidobacterium breve* strain Yakult, corn starch, hydroxypropyl cellulose (1 g)	corn starch and hydroxypropyl, cellulose	powder p.o. 3 times a day	powder p.o. 3 times a day	4–20
**Radiotherapy treatment**										
Shu-Xu Du et al. [37]	2018	China	NRS	80	80	*Bacillus licheniformis*	N/A	capsule p.o. 3 times a day	N/A	from the start of RT to the end of RT

^1^ RDBPC—randomized, double-blind, placebo-controlled; ^2^ CFU—colony-forming unit; ^3^ p.o.—orally; ^4^ RCT—randomized controlled study; ^5^ RT—radiation therapy; ^6^ NRS—non-randomized controlled study.

**Table 2:** Characteristics of the study population (*n* = 2619).

Study	Age (Years, Mean ± SD)		Sex (%Male)		Pathology (Patients, %)		Stage (Patients, %)			Chemotherapy	Radiotherapy (Total Dose, Gy)	Other Therapy (Patients, %)	Operation (Patients, %)
	Study	Control	Study	Control	Study	Control	Study	Control					
**ADULT POPULATION**													
**Chemotherapy treatment**													
Mego M. et al. [24]	62 (median) 45–75 (range)	64 (median) 42–81 (range)	60.9	52.2	colon carcinoma 69.6 rectal carcinoma 30.4		N/A	N/A		study (percentage of patients): irinotecan weekly 60.9 irinotecan every 2 or 3 weeks 39.1 5-fluorouracil 52.2 capecitabine 0 control (percentage of patients): irinotecan weekly 60.9 irinotecan every 2 or 3 weeks 39.1 5-fluorouracil 52.2 capecitabine 8.7	N/A	antiemetics, analgesics study: cetuximab 17.4 bevacizumab 26.1 control: cetuximab 21.7 bevacizumab 30.4	study: resection of the primary tumor 65.2 colostomy 34.8 control: resection of the primary tumor 82.6 colostomy 34.8
Liu J. et al. [36]	62.1 ± 10.9	60.1 ± 9.9	68		gastric cancer colorectal cancer lung cancer lymphoma		N/A	N/A		CHOP regimen: cyclophosphamide 750 mg/m^2^ i.v. ^1^ 1 day, hydroxy daunorubicin 50 mg/m^2^ i.v. 1 day, oncovin 1.4 mg/m^2^ i.v. 1 day, prednisone 40 mg/m^2^ p.o. 1–5 days TP regimen fluoropyrimidine-based chemotherapy regimen	No	N/A	N/A
**Radiotherapy treatment**													
Urbancsek H. et al. [22]	59	60	25	26	uterus cancer ovaries cancer prostate cancer rectum cancer lower abdomen cancer		N/A	N/A		No	50 (median) about 2 Gy daily	loperamide	N/A
Mansouri-Tehrani H.S. et al. [32]	63.73 ± 15.09	64.17 ± 11.69	67.4		colon and rectum 9 prostate 9 endometrium 4.5 bladder 6 ovary 3 cervical 1.5		colon and rectum 13.4 prostate 9 endometrial 3 bladder 6 ovary 1.5 cervical 3	N/A	N/A	N/A	40–50 1.8 Gy/day with 18 MV five fractions weekly for 4–5 weeks	N/A	N/A
Delia P. et al. [23]	N/A	N/A	N/A	N/A	sigmoid cancer rectal cancer cervical cancer		N/A	N/A		No	60–70	N/A	surgery for sigmoid, rectal or cervical cancer
Delia P. et al. [29]	range 45–65		51		colorectal carcinoma 53 cervical carcinoma 47		N/A	N/A		No	60–70	N/A	surgical anterior resection 53 hysterectomy 47
Delia P. et al. [31]	N/A	N/A	N/A	N/A	sigmoid cancer rectal cancer cervical cancer		N/A	N/A		No	adjuvant postoperative	loperamide	surgery for sigmoid, rectal or cervical cancer
Shao F. et al. [33]	60.2		48		abdominal tumour		N/A	N/A		N/A	<60	glutamine enteric capsule (0.25 g) p.o. 2 capsules 3 times a day fish oil soft capsule (1200 mg) p o. 3 times a day Peptisorb mixed with water	N/A
**Radiotherapy and chemotherapy treatment**													
Giralt J. et al. [18]	60.91 ± 11.80	59.34 ± 12.77	0		endometrial adenocarcinoma cervical squamous cell carcinoma		N/A	N/A		cisplatin i.v. 40 mg/m^2^ weekly (11 SG and 14 CG)	45–50.4 dose of 1.8–2 Gy/d, five times weekly for 5–6 weeks brachytherapy 2–3 weeks later	5-HT3 inhibitors	associated with cancer therapy
Linn YH. et al. [19]	57.38 ± 10.75	52.5 ± 9.61	0		squamous cell carcinoma adenocarcinoma anaplastic carcinoma cervical cancer		I B 7.7 II A 7.7 II B 46.2 III A 7.7 III B 26.9 IV A 3.8	I B 14.3 II A 3.6 II B 50 III A 14.3 III B 14.3 IV A 3.6		N/A	50.77 ± 2.72 study group 51.16 ± 3.43 Control group	N/A	study group 15 control group 14
Österlund P. et al. [28]	61	57	52	48	colorectal cancer		Dukes‘ stage B 28 C 56 Da16	Dukes‘ stage B 25 C 60 Da 15		levoleucovorin: 10/20 mg/m^2^ 5-FU: 370–425 mg/m^2^ i.v. bolus on days 1–5 of the cycle, repeated at 4-week intervals for six times 2-h infusion of levoleucovorin 200/400 mg/m^2^ followed by 5-FU 400 mg/m^2^ administered as an intravenous bolus and 48-h infusion of 3.0–3.6 g m−2 5-FU; this cycle was repeated every 14 days for 12 times 24 weeks	50.4 1.8 Gy daily, 5.5 weeks	11 g guar gum metoclopramide, 5-HT3 inhibitors, dexpanthenol lozenges 100–200 mg 3 times a day, pyridoxine 50 mg 3 times a day	associated with cancer therapy
Sharma A. et al. [20]	52.35 ± 9.433	50.09 ± 10.038	93		HNSCC ^2^ nasopharynx 10.9 oropharynx 47.5 hypopharynx 28.7 larynx 11.9	HNSCC nasopharynx 11.1 oropharynx 50.5 hypopharynx 28.3 larynx 9.1 others 1.0	I 2.97 II 5.9 III 44.6 IV 46.5	I 5.1 II 4.0 III 41.4 IV 49.5		cisplatin 40 mg/m^2^ weekly for 7 doses at 5 fractions per week	70 in 35 fractions over 7 weeks	analgesics study group 30 control group 45	N/A
Chitapanarux I. et al. [21]	47	52	N/A		squamous cell carcinoma of cervix		FIGO IIB 53.1 IIIB 46.9	FIGO IIB 58.1 IIIB 41.9		cisplatin 40 mg/m^2^ weekly for 6 weeks	56 200 cGy per fraction, five fractions per week brachytherapy: 28, Iridium-192 700 cGy per fraction, 4 insertions	loperamide (2 mg)	No
Topuz E. et al. [34]	51	58	64.86		colon cancer 35.3 rectosigmoid cancer 64.7	colon cancer 55.0 rectosigmoid cancer 45.0	ECOG III 82.4 IV 5.9 unknown 11.8	ECOG II 35 III 50 IV 15		median 6 cycles FOLFOX: folinic acid, 5-FU and oxaliplatin FUFA: folinic acid, 5-fluorouracil	adjuvant chemo-radiotherapy	N/A	No
de Sanctis V. et al. [30]	58.4 range (34–74)	60 range (39–77)	77.9		head and neck carcinoma		IIA 6.3 III 15.6 IV A 3.1 IV B 9.4	II A 0 III 13.9 IV A 66.7 IV B 11.1		cisplatin-based 40 mg/m^2^ weekly or 100 mg/m^2^ 3-weekly neoadjuvant chemotherapy (docetaxel, cisplatin and 5-fluorouracil every 21 days for three cycles (patients with nasopharyngeal cancer)	68–70 IMRT (macroscopic disease—intensity-modulated radiation therapy) 50–54 (low-risk regions)	cetuximab, biweekly	N/A
Jiang C. et al. [25]	51.69 ± 9.79	50.40 ± 10.25	63.79	60.00	nasopharyngeal carcinoma		T1 1.72 T2 17.24 T3 39.66 T4 41.38	T1 2.86 T2 8.57 T3 37.14 T4 51.43		cisplatin (100 mg/m^2^) three times during trial	70 in 32 fractions 2.19 Gy/d, 5 d/w; gross tumour volume) 60 in 32 fractions for 45 days; clinical target volume	oral cavity fungal infections: antifungal agents, soda water	N/A
Demers M. et al. [26]	Standard dose 61.4 High dose 62.0	60.6	standard dose 72 high dose 66	63	standard dose: prostate 32 endometrium 32 cervix 10 rectum 45 others 1 high dose: prostate 37 endometrium 8 cervix 7 rectum 41 others 7	prostate 30 endometrium 12 cervix 16 rectum 41 others 1	N/A	N/A		cervical cancers, cisplatin 40 mg/m^2^ rectal cancers, either 5-fluorouracil 225 mg/m^2^ in continuous perfusion or capsules of capecitabine (Xeloda) 825–1000 mg/m^2^ during the entire radiotherapy treatment	40–50.4 brachytherapy	N/A	N/A
Xia C. et al. [27]	range 18–70		N/A	N/A	nasopharyngeal carcinoma		N/A	N/A		cisplatin (100 mg/m^2^) on days 1, 22 and 43	32 fractions of 70 Gy radiotherapy (2.19 Gy/d, 5 d/wk) 32 fractions for 45 days (6–7 weeks in total)	N/A	N/A
**CHILD POPULATION**													
**Chemotherapy treatment**													
Reyna-Figueroa J. et al. [38]	10.8	10.7	63.3		acute lymphoblastic leukemia acute myeloblastic leukemia		high risk—56.7 usual risk—43.3	high risk— 60 usual risk— 40		prednisone p.o. 60 mg/m², 0 to 28 days; vincristine i.v. 2 mg/m², on days 0, 7, 14, 21, 28; daunorubicin i.v. 30 mg/m², on days 0 and 14; L-asparagine i.m. 10,000 UI/m² on days 5, 8, 12, 15, 19, 22;	No	N/A	No
Wada M. et al. [35]	6.5	7.25	40		acute lymphoblastic leukemia 33.3 non-Hodgkin lymphoma 33.3 yolk sac tumor 22.2 Ewing sarcoma 11	acute lymphoblastic leukemia 50 acute myeloid leukemia 9.1 non-Hodgkin lymphoma 18.2 Hodgkin disease 9.1 primitive neuroectodermal tumor 9.1 leiomyosarcoma 4.5	N/A	N/A		N/A	No	polymyxin B sulfate and sulfamethoxazole-trimethoprim granulocyte colony stimulating factor	N/A
**Radiotherapy treatment**													
Du S. et al. [37]	7.0	7.5	62.5	72.5	medulloblastoma 37.5 glioblastoma 30.0 ependymoma 21.2 astrocytoma 11.3	medulloblastoma 37.5 glioblastoma 30.0 ependymoma 21.2 astrocytoma 11.3	N/A	N/A		No	36 (CSI; range from 21 to 54 Gy) 1.5 (posterior fossa boost as; range from 1.5 to 1.8 Gy)	N/A	associated with cancer therapy

^1^ i.v. intravenous; ^2^ HNSCC head and neck squamous cell carcinoma.

**Table 3:** The occurrence of diarrhoea during the probiotic treatment.

Study	Grade (Percentage of Patients, %)						Duration (Days, Mean ± SD)		Frequency (Daily Incidents, Mean ± SD)		Consistency of Stool (Bristol Scale, Mean)		Abdominal Pain (Percentage of Patients, %)		Antidiarrheal Drug Used		General Result ^1^
	Study Group			Control Group			Study Group	Control Group	Study Group	Control Group	Study Group	Control Group	Study Group	Control Group	Study Group	Control Group	
**ADULT POPULATION**																	
**Chemotherapy treatment**																	
Mego M. et al. [24]	CTCAE ^2^ 4.1 1–21.7 2–17.4 3–0 4–0			CTCAE 4.1 1–34.8 2–8.7 3–13 4–4.3			N/A	N/A	N/A	N/A	N/A	N/A	N/A	N/A	loperamide 5.9 (mean tablets), diphenoxylate/atropine—0.3 (tablets)	loperamide—37.7 (mean tablets), diphenoxylate/atropine—21.3 (tablets)	non-significant
**Radiotherapy treatment**																	
Urbancsek H. et al. [22]	Investigator’s scale ^3^ mean grade 0.7			Investigator’s scale ^3^ mean grade 1.0			N/A	N/A	2.4	3.2	Investigators’ scale ^4^ 0.7	Investigators’ scale ^4^ 1.0	N/A	N/A	loperamide (35% patients; mean time to use 138 h)	loperamide (48% patients; mean time to use 125 h)	non-significant
Mansouri-Tehrani H.S. et al. [32]	NCI CTC ^5^ 2.0 2 or 3–31.8			NCI CTC 2.0 2 or 3–70.8			N/A	N/A	0–7 (range)	0–10 (range)	4.3	5.7	blounting 86.4	blounting 41.7	drug not specified (9.1% patients)	drug not specified (37.5% patients)	improvement
Delia P. et al. [23]	WHO ^6^ degrees 3 or 4–1.4			WHO degrees 3 or 4–55.4			N/A	N/A	5.1 ± 3	14.7 ± 6	N/A	N/A	N/A	N/A	loperamide (mean time to use 122 ± 8 h)	loperamide (mean time to use 86 ± 6)	improvement
Delia P. et al. [29]	WHO degrees 1–10 2–21 3–3 4–0			WHO degrees 1–10 2–12 3–17 4–13			N/A	N/A	N/A	N/A	N/A	N/A	N/A	N/A	N/A	N/A	N/A
Delia P. et al. [31]	scale not specified 1 or 2–30.53 3 or 4–7.37			scale not specified 1 or 2–21.05 3 or 4–29.47			N/A	N/A	4.6 ± 2	12.3 ±4	N/A	N/A	N/A	N/A	loperamide (mean time to use 118 ± 6 h)	loperamide (mean time to use 97 ± 4 h)	improvement
Shao F. et al. [33]	N/A			N/A			N/A	N/A	N/A	N/A	N/A	N/A	7 days after RT ^7^: 33.3 14 days after RT: 20.4	7 days after RT: 68.2 14 days after RT: 54.5	enteral nutrition, parenteral nutrition 17% patients	enteral nutrition, parenteral nutrition 64% patients	improvement
**Radiotherapy and chemotherapy treatment**																	
Giralt J. et al. [18]	NCI CTC 3.0 ≥ 3–45.45			NCI CTC 3.0 ≥ 3–36.59			N/A	N/A	N/A	N/A	5.95	5.94	N/A	N/A	loperamide (2 mg)		non-significant
Linn Y.H. et al. [19]	CTCAE 4.0 ^4^ 1 or 2–53.8 3 or 4–0			CTCAE 4.0 1 or 2–82.1 3 or 4–17.9			N/A	N/A	N/A	N/A	N/A	N/A	CTCAE 4.0 1–73.1 2–3.8 3.63 days ± 2.29	CTCAE 4.0 1–92.9 2–57.1 3–10.7 7.77 days ± 4.76	loperamide (50% patients; mean time to use 20.92 days)	loperamide (85.7% patients; mean time to use 18.04 days)	improvement
P. Österlund et al. [28]	NCI CTC 2.0 0 to 2–78 3 or 4–25			NCI CTC 2.0 0 to 2–63 3 or 4–37			N/A	N/A	N/A	N/A	N/A	N/A	discomfort: 59	discomfort: 75	loperamide	loperamide	improvement
Chitapanarux I. et a [21]	NCI CTC 2.0 1–55 2 or 3–45			NCI CTC 2.0 1–91 2 or 3–9			N/A	N/A	N/A	N/A	1–4 3% 5–6 78% patients 7 19%	1–4 0% 5–6 35% patients 7 65%	N/A	N/A	loperamide (2 mg; 9% patients)	loperamide (2 mg; 32% patients)	improvement
Demers, M et al. [26]	Control		Standard dose		High dose		N/A		Standard dose: 2.7, high dose: 2.8	2.9	standard dose: median 1.4 (1.2–1.8) high dose: median 1.5 (1.2–1.8)	median 1.6 (1.2–1.9)	NCI CTC 3.0 <1-100		loperamide (standard-dose 30.2% patients, high-dose 27.4% patients)	loperamide (42.5% patients)	non-significant
	WHO degrees with pelvic surgery 0–0 1–3.5 2–51.7 3–17.2 4–27.6 without pelvic surgery 0–10.5 1–19.3 2–47.4 3–21.1 4–1.8 total 87		WHO degrees with pelvic surgery 0–6.3 1–15.6 2–53.1 3–21.9 4–3.1 without pelvic surgery 0–20.4 1–26.5 2–40.8 3–10.2 4–1 2.0 Total 81		WHO degrees with pelvic surgery 0–16.7 1–5.6 2–38.9 3–27.8 4–11.1 without pelvic surgery 0–17.1 1–22 2–43.9 3–12.2 4–4.9 Total 59												
**CHILD POPULATION**																	
**Chemotherapy treatment**																	
Reyna-Figueroa J. et al. [38]	no case of diarrhoea			N/A			no case of diarrhoea	up to 5	no case of diarrhoea	N/A	no case of diarrhoea	N/A	no case of diarrhoea	N/A	no case of diarrhoea	N/A	improvement
Wada M. et al. [35]	N/A			N/A			1.06 ± 1.80	3.00 ± 3.84	0.5 ± 0.62	0.95 ± 0.79	N/A	N/A	N/A	N/A	polymyxin B sulphate and sulfamethoxazole-trimethoprim	polymyxin B sulphate and sulfamethoxazole-trimethoprim	non-significant
**Radiotherapy treatment**																	
Shu-Xu Du et al. [37]	CTCAE 3.0 1–14.3 2–42.9 3–42.9 4–0			CTCAE 3.0 1–10 2–50 3–40 4–0			N/A	N/A	N/A	N/A	N/A	N/A	N/A	N/A	N/A	N/A	improvement

^1^ Significantly better outcomes in the study group in at least one parameter (α = 0.05); ^2^ CTCAE—Common Terminology Criteria for Adverse Events; ^3^ 0 none, 1 mild, 2 moderate, 3 severe; ^4^ 0 normal, 1 soft or malformed, 2 pasty, 3 liquid stools; ^5^ NCI CTC—National Cancer Institute Common Toxicity Criteria; ^6^ WHO—World Health Organisation; ^7^ RT—radiotherapy treatment.

**Table 4:** The occurrence of constipation during the probiotic treatment.

Study	Duration (Days)		Frequency and Character (Percentage of Patients, %)		Wexner Score (Percentage of Patients, %)		Gener Result ^1^
	Study Group	Control Group	Study Group	Control Group	Study Group	Control Group	
**ADULTS POPULATION**							
**Chemotherapy treatment**							
Liu J. et al. [36]	N/A	N/A	Markedly ^2^: 18 Effective ^3^: 78 Invalid ^4^: 4	Markedly: 8 Effective: 24 Invalid: 68	0–10: 37 11–20:13 21–30:0	0–10: 35 11–20: 15 21–30: 0	improvement
**CHILD POPULATION**							
**Chemotherapy treatment**							
Reyna-Figueroa J. et al. [38]	up to 5	up to 7	N/A	N/A	N/A	N/A	improvement

^1^ Significantly better outcomes in the study group in at least one parameter (α = 0.05); ^2^ Markedly: stool returned to normal and the frequency to once a day after treatment; ^3^ Effective: stool character improved and the frequency became more than 3 times per week after treatment; ^4^ Invalid: no improvement in frequency and character of stool after treatment.

**Table 5:** The occurrence of nausea and vomiting during the probiotic treatment.

Study	Duration of Vomiting (Days)		Duration of Nausea (Days)		Nausea Grade (CTCAE ^2^ 3.0, Percentage of Patients, %)		Vomiting Grade (CTCAE 3.0, Percentage of Patients, %)		General Result ^1^
	Study Group	Control Group	Study Group	Control Group	Study Group	Control Group	Study Group	Control Group	
**CHILDREN POPULATION**									
**Chemotherapy treatment**									
Reyna-Figueroa J. et al. [38]	up to 6	up to 7	up to 7	up to 7	N/A	N/A	N/A	N/A	improvement
**Radiotherapy treatment**									
Shu-Xu Du et al. [37]	N/A	N/A	N/A	N/A	I 16.25 II 30 III 16.25 IV 3.75	I 12.5 II 36.25 III 26.25 IV 7.5	I 6.25 II 16.25 III 12.5 IV 35	I 7.5 II 26.25 III 16.25 IV 2.5	improvement

^1^ Significantly better outcomes in the study group in at least one parameter (α = 0.05); ^2^ CTCAE—Common Terminology Criteria for Adverse Events.

**Table 6:** The occurrence of mucositis during the probiotic treatment.

Study	Localization of Mucositis	Grade (Percentage of Patients, %)		Time to Onset of Mucositis (Days)		Time to Resolution or Healing (Days, Median)		Administration of Additional Nutrition (Percentage of Patients, %)		General Result ^1^
		Study	Control	Study	Control	Study	Control	Study	Control	
**ADULT POPULATION**										
**Radiotherapy and chemotherapy treatment**										
de Sanctis V. et al. [30]	oral cavity	CTCAE ^2^ 4.0 III or IV— 40.6	CTCAE 4.0 III or IV— 41.6	N/A	N/A	N/A	N/A	enteral nutrition 37.5	enteral nutrition 16.6	non-significant ^3^
Sharma A. et al. [20]	oral cavity	NCI CTC ^4^ 2.0 0—28 I—11 II—8 III—2 IV—50	NCI CTC 2.0 0—7 I—10 II—5 III—8 IV—69	22 (±13.2)	18 (±11.6)	43	43	parenteral nutrition or insertion of a Ryle’s tube 22	parenteral nutrition or insertion of a Ryle’s tube 34	improvement
Topuz E. et al. [34]	oral cavity	NCI CTC 2.0 0 72.7 I 12.1 II 12.1 III 1.0 IV 2.0	NCI CTC 2.0 0 78.3 I 13.2 II 7.5 III 0.9	N/A	N/A	N/A	N/A	N/A	N/A	non-significant
Jiang C. et al. [25]	oral cavity	CTCAE 4.0 0—12.07 I—55.17 II—17.24 III—15.52	CTCAE 4.0 0—0 I—0 II—54.29 III—45.71	N/A	N/A	N/A	N/A	N/A	N/A	improvement
Xia C. et al. [27]	oral cavity	CTCAE 4.0 0—13.9 I—36.1 II—25 III—22.2 IV—2.8	CTCAE 4.0 0—0 I—14.7 II—38.2 III—32.4 IV—14.7	N/A	N/A	N/A	N/A	N/A	N/A	improvement
**CHILD POPULATION**										
**Radiotherapy treatment**										
Shu-Xu Du et al. [37]	oral cavity	CTCAE 3.0 I—66.7 II—33.3 III—0 IV—0	CTCAE 3.0 I—31.8 II—45.45 III—22.7 IV—0	N/A	N/A	N/A	N/A	N/A	N/A	improvement

^1^ Significantly better outcomes in the study group in at least one parameter (α = 0.05); ^2^ CTCAE—Common Terminology Criteria for Adverse Events; ^3^ significant—in need of enteral nutrition for patients in experimental group compared to control group; ^4^ NCI CTC—National Cancer Institute Common Toxicity Criteria.

## Data Availability

Not applicable.
